# Electrosensory Contrast Signals for Interacting Weakly Electric Fish

**DOI:** 10.3389/fnint.2019.00036

**Published:** 2019-07-31

**Authors:** Na Yu, Ginette Hupe, André Longtin, John E. Lewis

**Affiliations:** ^1^Department of Mathematics and Computer Science, Lawrence Technological University, Southfield, MI, United States; ^2^Department of Physics, University of Ottawa, Ottawa, ON, Canada; ^3^Department of Biology, University of Ottawa, Ottawa, ON, Canada; ^4^Brain and Mind Research Institute, University of Ottawa, Ottawa, ON, Canada

**Keywords:** electrosensation, envelope, contrast, dichotomous noise, Hilbert transform, swimming behavior, active sensing

## Abstract

Active sensory systems have evolved to properly encode natural stimuli including those created by conspecifics, yet little is known about the properties of such stimuli. We consider the electrosensory signal at the skin of a fixed weakly electric fish in the presence of a swimming conspecific. The dipole recordings are obtained in parallel with video tracking of the position of the animals. This enables the quantification of the relationships between the recording dipole and the positions of the head, midbody and tail of the freely swimming fish. The contrast of the signal at the skin is shown to be well-fitted by a decreasing exponential function of distance. It is thus anti-correlated with distance; it is also correlated with the second envelope (i.e., the envelope of the envelope) of the raw recorded signal. The variance of the contrast signal is highest at short range. However, the coefficient of variation (CV) of this signal increases with distance. We find a range of position and associated contrast patterns under quasi-2D swimming conditions. This is quantified using global measures of the visit times of the free fish within measurable range, with each visit causing a bump in contrast. The durations of these bumps as well as the times between these bumps are well reproduced by a doubly stochastic process formed by a dichotomous (two-state) noise with Poisson statistics multiplying a colored noise [Ornstein-Uhlenbeck (OU) process]. Certain rapid body movements such as bending or turning are seen to produce contrast drops that may be part of cloaking strategies.

## Introduction

Wave-type weakly electric fish generate electric organ discharges (EODs) to sense their environment and communicate with conspecifics in the dark. Individual fish have a signature EOD (carrier) frequency. Amplitude modulations (AMs) of this carrier provide sources of sensory information: the frequency and phase content of AMs (i.e., beat frequency) can provide information about the EOD frequency and identity of conspecifics (Yu et al., 2012; Shifman and Lewis, 2018); and the amplitude content of AMs (i.e., contrast or second envelope) can represent motion and conspecific location (Yu et al., 2012; Fotowat et al., 2013). The focus of this article is on the latter, i.e., the relationship between contrast and motion. In particular, we study how the relative motion of two interacting fish affects the EOD modulations that provide sensory information about conspecific location. Motion perception is influenced by both object motion and observer motion. As a first step, we consider an intermediate situation in which one fish (the observer) is restrained to a stationary mode while another fish (the object) swims freely. This allows us to characterize the specific contributions of object (conspecific) motion to the contrast in EOD modulations. The associated signal is also relevant to the context in which a stationary fish in the wild, perhaps hiding in plants, images other fish swimming in its neighborhood.

Previous work has investigated how contrast signals vary with the distance between interacting electric fish (Yu et al., 2012; Fotowat et al., 2013; Metzen and Chacron, 2014). As expected from Coulomb’s Law and the dipole nature of the electric fields generated by these fish (Babineau et al., 2006), contrast signals fall off quickly with inter-fish distance. By recording transdermal potential in freely swimming fish, Fotowat et al. (2013) clearly demonstrated this general trend but also showed that additional factors (besides distance) have an influence on signal contrast. These factors include relative orientation and pose (degree of body bending) of the fish. Here, we describe these relationships in more detail, as well as swimming patterns and their associated contrasts as a function of the distance between a patch of skin on the fixed fish to the head, mid-body and tail of the moving fish. We also provide a quantitative description, in the form of a mathematical model, that reproduces the dynamics of contrast variations during our experiments. Such a model can be used to experimentally or computationally mimic the presence of a moving conspecific.

The article is organized as follows. In “Materials and Methods” section, we outline the experimental and computational methods used for our work. “Results” section describes the experimental results, their analysis and the proposed doubly stochastic model for interactions of two fish under the conditions of our experiment. The article ends with a discussion and outlook onto future work.

## Materials and Methods

### Experiments

This study was approved by the animal care committee of the University of Ottawa (BL-229; BL-1773) and carried out in accordance with the guidelines of Canadian Council on Animal Care. Mature male and female *A. leptorhynchus* were obtained from a tropical fish supplier. Fish were kept in large flow-through community tanks on a 12:12 h light:dark cycle with 0–4 tank mates, and fed thawed blood worms three times weekly. All experiments were performed within the first few hours of the dark phase of the light cycle in a tank measuring 30 × 40 cm with a depth of either 4 cm or 10 cm.

To characterize the contrasts produced while one fish is stationary and the other fish freely swims, we restrained one fish in a hammock (the restrained fish or reference fish, “Rfish,” which acts as an observer) in the center of the tank while a second fish (the free-swimming fish, “Ffish,” which acts as an observed object) was allowed to swim freely in the tank around it (Yu et al., 2012). All experiments were performed in the dark. The hammocks were created using rectangular tulle holders measuring 15 cm long and 6 cm deep, closed along the top edge with Velcro; while in these hammocks, the fish showed no signs of discomfort, and produced chirps readily. We recorded the electrical potential using a pair of electrodes attached to the hammock and positioned adjacent to the head of the Rfish near the operculum with the tips of the electrodes positioned 1 cm apart, perpendicular to the axis of the Rfish. The position of this pair of electrodes was chosen to sample the composite electrical image received near the rostral surface of the Rfish’s body (i.e., available to nearby electroreceptors) during conspecific movements. The recorded signals were primarily composed of the Rfish’s EOD, with the influence of the Ffish’s EOD increasing whenever the Ffish moved closer to the Rfish.

Electrical recordings sampled at 50 kHz were acquired using Teflon-coated silver wire electrodes, a differential amplifier (AM Systems, Sequim, WA, USA), and a D1104 A/D system (dSpace Inc., Wixom, MI, USA). Electrical recordings were collected from a total of 12 randomly chosen pairs of fish for 5 min. In four of these trials, we also videotaped the interactions using an infrared (IR) video camera positioned above the tank (with the tank illuminated from below using an IR light panel) to record physical behaviors of the Ffish over the course of the interaction while simultaneously acquiring electrical recordings.

### Data Analysis and Definitions

All data analyses and numerical simulations were carried out in MATLAB (The MathWorks, Inc., Natick, MA, USA). We calculated first- and second-order envelopes as well as instantaneous contrast time series (defined below) from electrical recordings in all 12 trials. The position and distance data (defined below) were calculated based on the video recordings of four fish pairs.

#### The First-Order Envelope (E1) and the Second-Order Envelope (E2)

**Figure 1:**
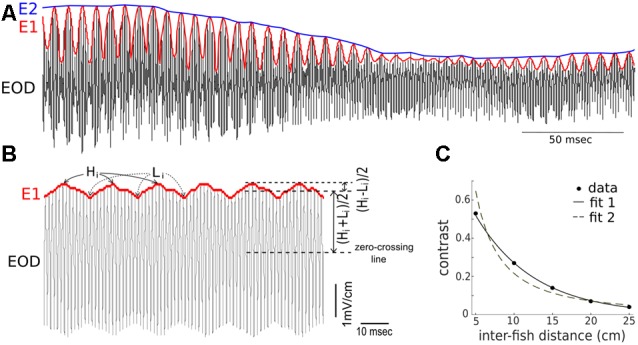
Summary of the terms used to describe the composite recorded electric signal produced during conspecific interactions. **(A)** Shown are depictions of the electric organ discharge (EOD; black), the first-order envelope (E1, red), and the second-order amplitude (E2, blue) in a signal recorded during an interaction of two fish (one restrained “Rfish” and one free-swimming “Ffish”). The frequency of the E1 modulation is a consequence of the difference in the EODf of the interacting fish. The magnitude of E2 is related to the distance separating the two fish, and E2 modulations result from changes in the position and orientation of the fish. **(B)** Instantaneous values of contrasts indicate the magnitude of E2 and are determined by calculating, over time, the size of the modulation depth relative to the amplitude of the recorded signal. The heights of the E1 peaks are denoted by H1, while the heights of the troughs are denoted by L1. “Contrast” = [(H_i_ − L_i_)/2]/[(H_i_ + L_i_)/2], and varies between 0 and 1. **(C)** When two fish are restrained oriented parallel to one another, an electrical recording sampled adjacent to the skin of one of the fish, shows the contrast increases as the distance between the fish decreases. The inverse relationship can be fitted as: contrast = 0.9977 *e*^−(13.04* distance)^ with *R*^2^ = 0.9989 (fit 1). It can also be fitted, although slightly less well, by a power law as: contrast = 0.56 (distance)^−1.586^ with *R*^2^ = 0.9555 (fit 2). Note that the parameters in the fitting curve could take different values for another pair of fish.

The AM (i.e., the first-order envelope, E1) of the recorded signal, *s* (i.e., EOD) can be calculated as $E1=\sqrt{s^{2}+{\hat{s}}^{2}}$, where ŝ is the Hilbert transform of *s*, i.e., $\hat{s}=\frac{1}{\pi t}*s$ where * denotes convolution. The AM of E1 (i.e., the second order envelope, E2) can also be obtained by applying the above method on E1, i.e., $E2=\sqrt{E1^{2}+{\hat{E1}}^{2}}$, where $\hat{E1}$ is the Hilbert transform of *E*1 Examples of E1 and E2 are shown in Figure 1A.

#### Instantaneous Contrast

**Figure 2:**
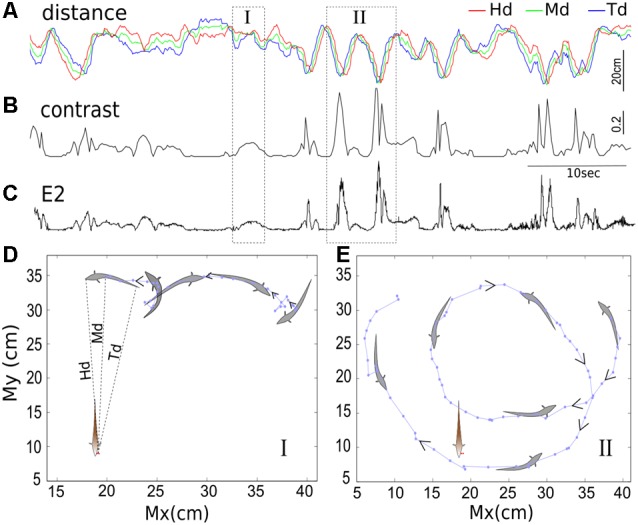
The temporal relationships between inter-fish distances **(A)**, contrast **(B)** and second envelope E2 **(C)**. The distance separating the free-swimming fish and the recording electrodes positioned next to the operculum of the restrained fish are depicted in the top trace. The red, green and blue lines indicate the distance from the recording electrodes to the free-swimming fish’s head, middle of the body, and end of the tail, denoted by Hd, Md and Td, respectively. The instantaneous contrast and E2 calculated over time are shown in the second and third traces, respectively; both are inversely correlated with inter-fish distance. The position of the free-swimming fish in epochs indicated by boxes I and II are shown in (**D,E**; in 100 ms increments), with the arrows indicating the direction of movement over time. During epoch I, the fish swims backward towards the recording electrodes and then changes direction, and this results in a contrast bump. In epoch II, the fish swims backward, looping twice around the tank, passing close to the recording electrodes each time. These movements are evident in the distance minima and corresponding contrast peaks. Each point indicates the position of the middle of the fish’s body (Mx, My) measured at 100 ms increments.

The sum of the two EODs at the recording dipole against a patch of skin on Rfish was recorded. From these recordings, the time-varying contrasts and envelopes were calculated. In the presence of one another, the fish each experience a beating EOD pattern (e.g., Yu et al., 2012). The beat frequency is equal to the difference between the individual EOD frequencies of the two fish. The amplitude of this beating pattern at the recording dipole is time-dependent, as it depends on the relative distance and orientation of the two fish (Kelly et al., 2008; Fotowat et al., 2013). This complicates the quantification of contrast. Instead of reporting a contrast at every sampling point of the EODs, we compute one contrast value per beat cycle using the following method. We collected the highest points and lowest points of E1. As shown in Figure 1B, *H_i_* and *L*_i_ denote *i-th* highest and lowest points, respectively. The “instantaneous” contrast during one beat cycle is defined by the ratio of the half-difference between *H*_i_ and *L*_i_ to the average of *H*_i_ and *L*_i_, that is, $\text{Contrast}=\frac{(H_{i}-L_{i})/2}{(H_{i}+L_{i})/2}$. This quantity ranges from 0 to 1, and is thus a dimensionless measure of contrast. Examples of instantaneous contrast are shown in Figure 2B. This peak-to-peak contrast is the same as the Michelson contrast used in the field of vision research (Michelson, 1927).

#### Representation of Video Recording

The position of the Ffish’s head, midbody, and tail was extracted with 100 ms resolution from the video recordings using Videopoint Capture tracking software. The three pairs of points (Hx, Hy), (Mx, My) and (Tx, Ty) are used to denote the coordinates of Ffish’s head, midbody and tail position in a 2D plane, respectively. The position of the electrodes beside the Rfish was also extracted from the video recording. We then calculated three measures of inter-fish distance (separating Ffish and Rfish): from the head, the midbody, and the tail of Ffish to the electrodes next to Rfish (Figure 2). These distances are denoted as HD, MD and TD, respectively.

## Results

### Instantaneous Contrast Is Anti-correlated With Inter-Fish Distance

When two fish are stationary (e.g., experimentally restrained with a fixed distance and parallel to one another), the contrast of the composite electrical signal that one fish receives (for example, at a skin position near our recording dipole) is a constant. The value of this constant depends on the distance between the animals and their relative orientation. The contrast vs. inter-fish distance is well-fitted from 5 cm to 25 cm by an exponential, as shown in Figure 1C.

When two fish freely swim, the dynamic inter-fish distance causes a time-varying contrast. Based on Coulomb’s law, we expect the EOD amplitude to drop off quickly with distance from the fish given the dipolar nature of the field. Thus, changes in the distance separating two fish (inter-fish distance) are expected to be negatively correlated with the EOD contrast. We examined the temporal relationship between the inter-fish distance, the instantaneous contrast and the second envelope E2. The inter-fish distance and the contrast generally exhibit the expected negative correlation (Figures 2A,B). We also first observe that the contrast and E2, although resulting from different computational methods as stated in “Materials and Methods” section, are both caused by the movement of Ffish, and are proportional to one another (Figures 2B,C). This observation is consistent with previous studies that fish movement can produce E2 (Yu et al., 2012; Fotowat et al., 2013; Metzen and Chacron, 2014), and specifically in an anti-correlated pattern (Stamper et al., 2013). Meanwhile, we are also aware of the fluctuations on the contrast and E2 (more obvious in E2). There are many factors that are able to generate contrast variability, for example, the occurrence of chirps, tail-bending and sudden changes in swimming orientation. Weakly electric fish tend to chirp more when they are in close proximity, often resulting in a transient (~50 ms) decrease in E1, and a further decrease in E2 and contrast (Hupé and Lewis, 2008; Henninger et al., 2018).

In order to better understand how the movement of Ffish is projected to the instantaneous contrast (or E2), we reconstructed the swimming trajectories of Ffish from video-recordings. Figures 2D,E highlight two brief epochs during one experiment (denoted I and II). During epoch I (Figure 2D), Ffish hovers laterally, then turns and swims in reverse before turning again and swimming forward in the same direction. Note that the swim reversal results in a small change in Hd, but an obvious change in Td (Figure 2A), along with a contrast peak (Figure 2B). During epoch II (Figure 2E), Ffish swims in reverse following a looping trajectory and passing by Rfish very closely twice, resulting in two major peaks in contrast and E2 (Figures 2B,C).

**Figure 3:**
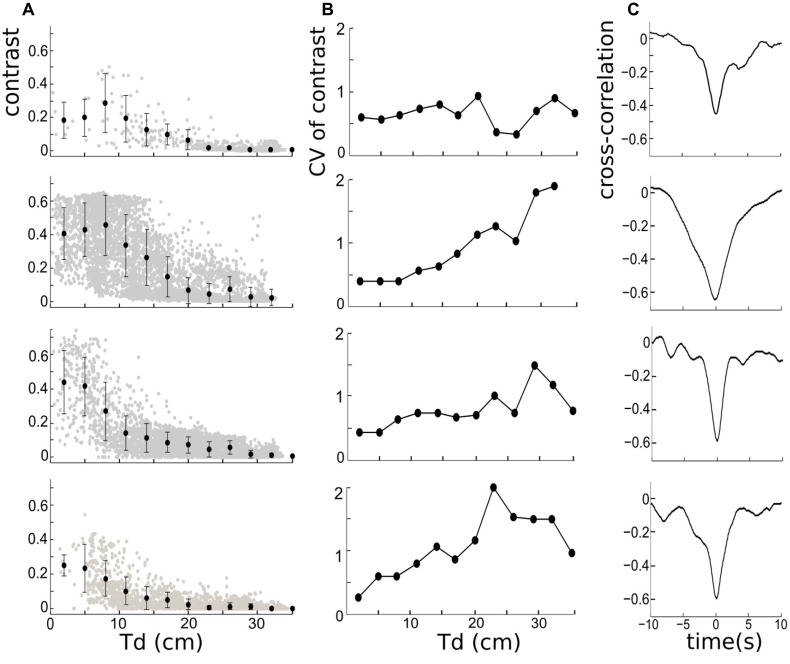
**(A)** Left column: the negative relationship between instantaneous contrast and inter-fish distances measured from the tail of the free-swimming fish to the electrodes (Td), for four trials over 300 s. The errors bars on the mean contrasts correspond to ± 1 standard deviation, and reveal that the contrast variability is larger when mean distances are small. The mean contrast is calculated based on the bin-width of 10/3 cm. **(B)** Middle column: the corresponding coefficient of variation (CV) of contrast for four trials. The CV is largest at larger distances for three out of the four cases shown. **(C)** Right column: the cross-correlation functions depicting the relationship between contrast and Td. The significant negative correlation is present in all trials. The width of the trough and shape of the correlation vary across trials.

The anti-correlation between inter-fish distance and instantaneous contrast was then quantified. The inter-fish distance is represented by Td here because the field strength is highest in the tail region (e.g., Shifman and Lewis, 2018). The mean of the instantaneous contrast associated with different Td (every 0.03 cm) decays with increasing Td for all four fish pairs (Figure 3A), which is consistent with our previous observation of their temporal relationship. This negative relationship can also be quantitatively measured by the cross-correlation, which shows rather large negative correlation coefficients (−0.4 to −0.65) for the four fish pairs (Figure 3C). Using other inter-fish distances (Hd or Md) leads to the same conclusion (data not shown). The width and depth of the cross-correlation trough vary over trials. The most active fish pair (Figure 3 second row—pair 2) has a higher contrast variability than other pairs, resulting in a larger width of the cross-correlation trough. We also found that the bottom of the trough, corresponding to the most negative correlation, occurs for 0 lag between the signals; and although the standard deviation of contrast appears to be higher when Td is small (Figure 3A), the relative variability of the contrast, measured by the coefficient of variation (CV, defined by the ratio of the standard deviation and the mean), is actually smaller when Td is smaller (Figure 3B). This suggests that changes in contrast may convey distance information even at relatively low absolute levels.

### Spatial Distribution of the Contrast

**Figure 4:**
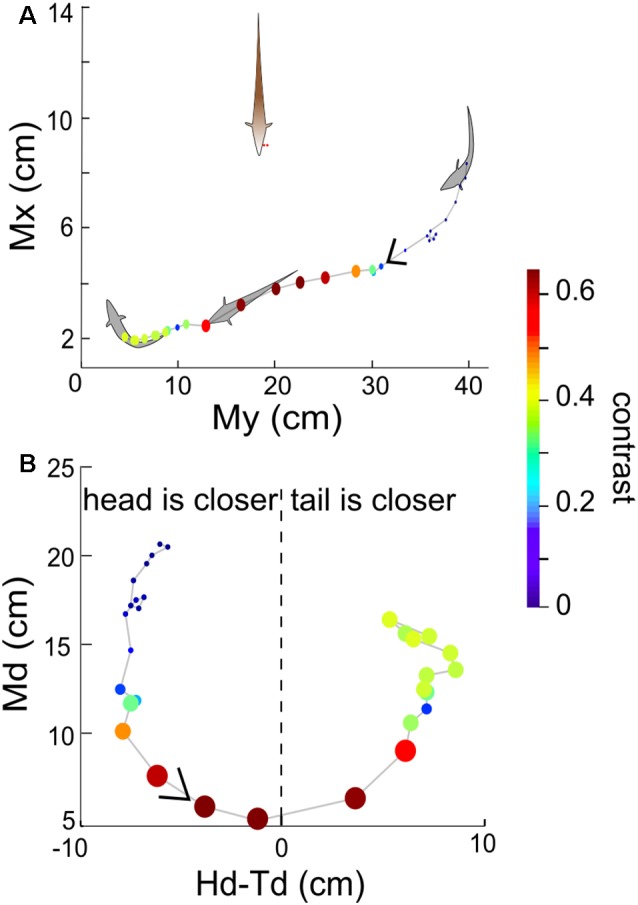
**(A)** Example recorded time course of free-swimming fish movements and the associated contrast in 3 s. Each point indicates Mx and My of the free-swimming fish, measured at 100 ms increments. The size and color of the point indicate the contrast at that instant. The arrow indicates the direction of movement over time. **(B)** Md vs. Hd-Td for the same behavioral sequence as shown in **(A)**. The value Hd-Td provides information about the orientation and curvature of the free-swimming fish relative to the recording electrodes. Negative (positive) values of Hd-Td correspond to the head of Ffish being closer to (farther from) the recording electrodes. The orientation of the fish’s body changes as the fish swims around and influences the calculated value of contrast.

**Figure 5:**
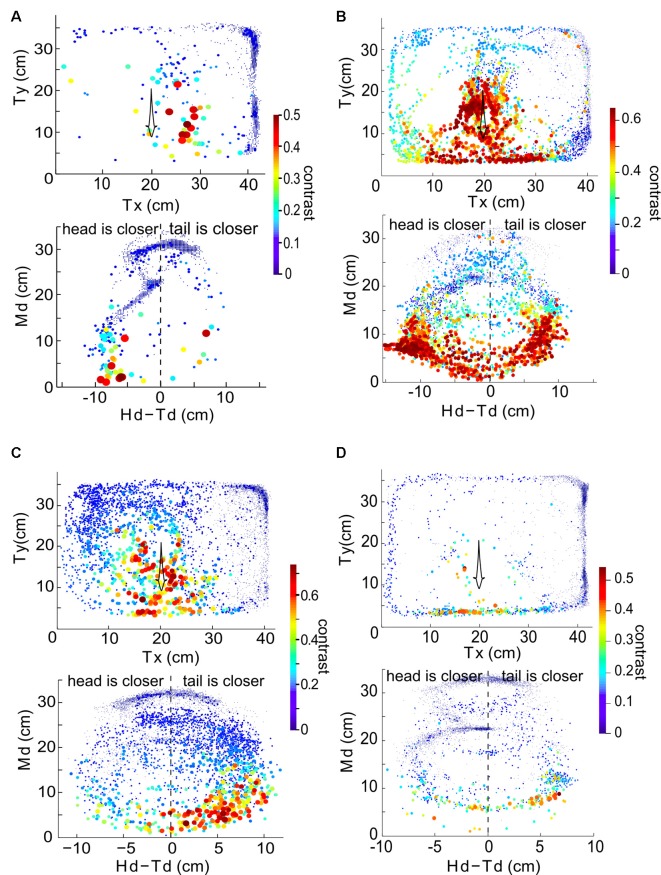
The relationship between instantaneous contrast and the position of the free-swimming fish in the experimental tank over 300 s for the same four fish as in Figure 3. Data from a given pair of fish is plotted in each of the four panels. Top row in each panel: Tx and Ty indicate the position of the tail of the free-swimming fish in the tank relative to the bottom left corner of the tank, in centimeters. Each point indicates the position of the fish (averaged over a 100 ms time bin), and the size and color of each point (as shown in the color bar) denotes the value of the instantaneous contrast determined at that point in time. Bottom row in each panel: Md plotted against Hd-Td reveal orientation information. Panel **(A)** uses data from the fish pair in the first row of Figure 3, panel **(B)** to the second row of Figure 3, panel **(C)** to the third row of Figure 3 and panel **(D)** to the fourth row of Figure 3.

We now consider the spatial distribution of the contrast during these interactions. In general, the contrast is high (≥0.3) when Ffish is within a radius of approximately 15 cm around Rfish (Figures 4, 5). As expected, the closer two fish are, the higher the contrast, but body movements can produce brief decreases. As a consequence, we then examined whether swimming orientation is also represented in the local contrast signals. Here swimming orientation is approximately measured by the difference between the distances from RFish to the head and tail of Ffish, i.e., Hd-Td. When two fish are in a static state, contrast is larger if the tail is closer to Rfish (positive “Hd-Td”). However, this is not always true in the free-swimming state (bottom rows in Figures 5A–D). This is because chirps, turning or tail-bending can easily change the contrast and further cause high variability in contrast. This suggests that the electric image over a large region of the fish body is needed to identify the orientation of a neighboring fish and trace their movement (Pedraja et al., 2018).

### Contrast Bumps and Their Temporal Distribution

**Figure 6:**
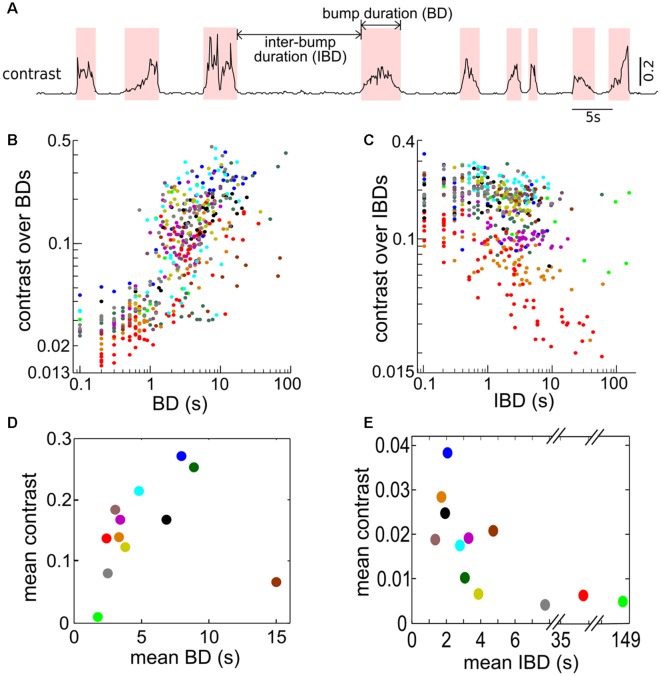
Bumps in contrasts are associated with approach behaviors. **(A)** Trace showing instantaneous contrast calculated for one recording over 70 s demonstrating the occurrence of contrast bumps. Contrast bumps were defined as any time period in which the contrast remained above a value of 0.04 for two or more consecutive time bins (i.e., for 200 ms or longer). The contrast bump and inter-bump intervals are denoted as bump duration (BD) and inter-bump duration (IBD), respectively. **(B)** The average contrast calculated over each BD vs. the corresponding BD. **(C)** The average contrast calculated over each IBD vs. the corresponding IBD. **(D)** The relationship between the mean contrast calculated over all BDs plotted against the mean BD (in seconds). Each dot indicates one fish pair; 12 fish pairs are used. In general, longer BIs (i.e., longer duration when free-swimming fish is in the proximity of restrained fish) are associated with larger contrasts except the brown dot representing a fish pair where they kept a certain distance quietly most of the time. **(E)** The relationship between the mean contrast calculated during IBDs plotted against the mean IBD. For longer IBDs, the free-swimming fish tends to be further away from the restrained fish, resulting in lower contrasts. Panels **(D,E)** are color coded as **(B,C)** for each pair of fish.

The time-varying contrast exhibits random occurrences of bumps because of the looming (i.e., approaching) and receding (i.e., departure) behaviors of Ffish (Figures 2A,B, 6A). Our working definition of contrast bump is any time interval during which the contrast remains over 0.04 for two or more consecutive 100 ms bins. We calculated the duration of the contrast bumps (bump duration, BD), the intervals separating two adjacent bumps (inter-bump duration or IBD), and the average of the instantaneous contrast within each BD and IBD for all interactions (6B,C). The contrast tends to increase with longer BDs but decrease with longer IBDs. To get a more comprehensive picture, we calculated mean BD, mean IBD and the means of all average contrasts over BDs and IBDs for each pair of fish (Figures 6D,E); each dot represents one pair of fish and is color-coded as in Figures 6B,C. This averaged contrast information indicates that longer BD and shorter IBD are related to higher mean contrast, for 11 out of 12 pairs. The brown dot in Figure 6D comes from a pair in which the Ffish was often relatively immobile at a certain distance from Rfish.

**Figure 7:**
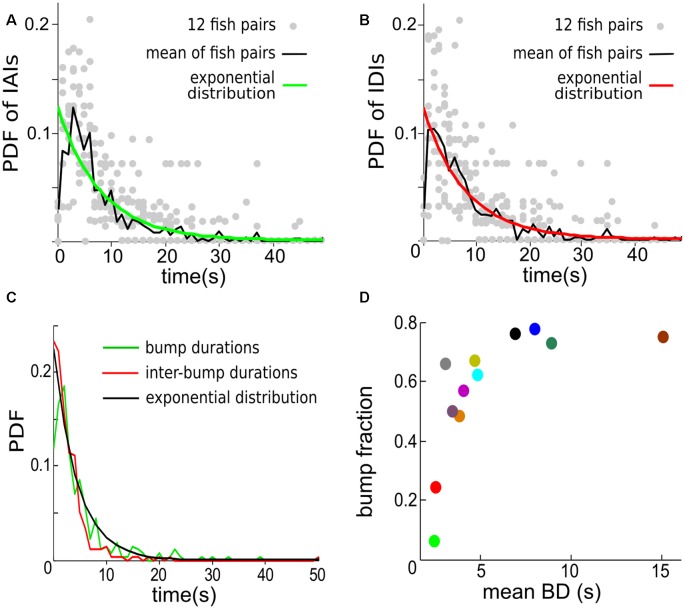
The temporal distribution of contrast bumps across 12 individual fish pairs. **(A)** The probability density functions (PDFs) for individual trials (gray dots) and for the mean calculated across trials (green curve) of the intervals separating the start times of consecutive contrast bumps, i.e., inter-“arrival” intervals (IAIs). The mean IAI calculated over trials is 8.07 s. The PDF of an exponential distribution with mean rate λ equal to 8.07 s (black curve) is shown to fit well the observed mean IAI density. **(B)** The PDF for individual trials (gray dots) and for the mean calculated across trials (red curve) of the intervals separating the end times of consecutive contrast bumps, i.e., inter-departure intervals (IDIs). The mean IDI across trials is 8.18 s. The PDF of an exponential distribution with λ equal to 8.18 s (black curve) is again in good agreement with the observed mean IDI density. **(C)** The mean PDF of BDs and IBDs. The mean BD is 3.2 ± 0.7 s. An exponential distribution with λ equal to 4.44 s approximates the density of both BDs and IBDs. A bin width of 1 s is used for estimating the PDFs. **(D)** The bump fraction (fraction of time the contrast spends above chosen value of 0.04) vs. the mean BD for each fish pair. The data are color coded as in Figures 6D,E.

The start and end of a contrast bump reflect the “arrival” and “departure” respectively of Ffish, therefore contrast bumps can be used to characterize the interaction times between fish. The mean probability density function (PDF) of inter-arrival intervals (IAIs) averaged over 12 fish pairs is found to be well fitted by an exponential distribution with mean of 8.07 s (Figure 7A). Similarly, the mean inter-departure intervals (IDIs) is also well fitted to an exponential process with mean of 8.18 s, which is also the mean of IDIs (Figure 7B). The statistical analysis indicates that the looming and receding events of Ffish occur in a random pattern over time, which can be approximately described by Poisson processes with means 1/8.07 s and 1/8.18 s, respectively. Figure 7C shows that BDs and IBDs are again well characterized by an exponential distribution with 4.4 s and here the mean BD is 3.2 ± 0.7 s.

We then defined the Bump Fraction as the sum of all BDs divided by the trial time. A larger bump fraction implies that the Ffish stays at relatively close range to Rfish for a longer time. But a large bump fraction does not necessarily lead to a high average contrast; comparing Figure 7D with Figure 6B, one sees for example that large bump fractions can occur for a mean BD around 5 s (Figure 7D), which correspond only to contrasts around 0.2 (Figure 6B). This is due in part to the threshold value of 0.04 that we used to define a contrast bump.

### Stochastic Model for Long-Term Dynamic Contrast

We next seek a quantitative description with which to model the movement patterns under the conditions of our experiments. Such a quantitative description (i.e., simulated signal) has general usages in various experimental paradigms, including creating an artificial weakly electric fish capable of mimicking naturalistic signals, producing pseudo-natural stimuli to study neuronal processing and simulating natural inputs to computational models of the sensory pathway. The goal is to quantify the stochastic movement patterns with a small number of stochastic processes and parameters.

Our results so far enable us to extend the model of long-term instantaneous contrasts associated with movement that we developed in a previous study of movement encoding using envelopes (Yu et al., 2012). Specifically, we can write:

(1) Contrast=ξ(A+ση)

where we smooth out the variations from beat cycle to beat cycle, i.e., the contrast is a coarser representation of the movement envelope. In our previous study, ξ was simply a constant factor. Here however, to account for the looming and receding activities that lead to contrast bumps, we define ξ as a dichotomous noise, i.e., a two-state noise with Poisson-distributed (i.e., exponentially distributed) residence time in each state. It is also referred to as random telegraph noise. This dichotomous noise describes the random switching between contrast bump states (the high state of the dichotomous noise) and inter-bump states (the low state). The parameters A and σ are the mean and standard deviation of contrast bumps; η is Ornstein-Uhlenbeck process (OU) which is a simple type of lowpass-filtered Gaussian white noise with a cutoff frequency equal to the reciprocal of the autocorrelation time. The OU process has an exponentially decaying auto-correlation function, with correlation time of 1 s (Yu et al., 2012); it generates the fluctuations within contrast bump intervals.

**Figure 8:**
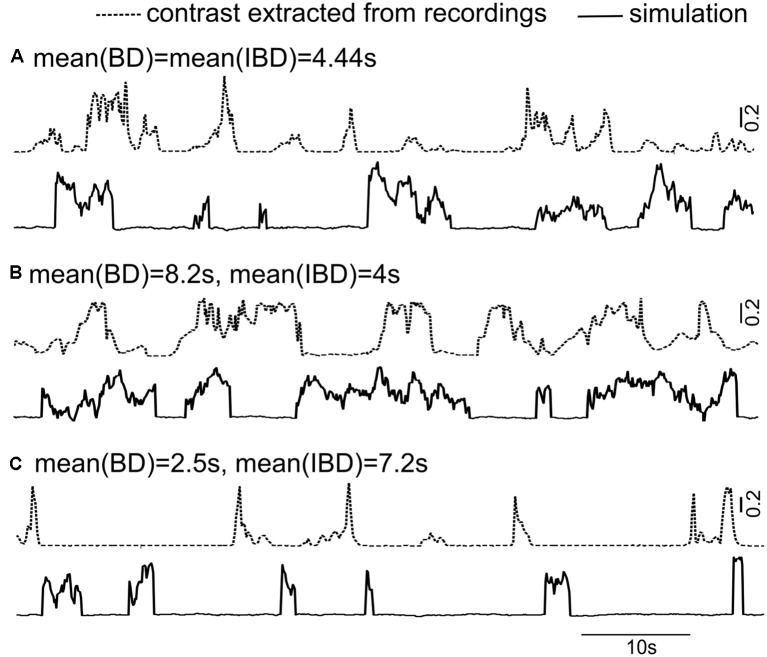
The doubly stochastic model Contrast = ξ (*A* + *ση*) (see Equation 1) is used to simulate the long-term instantaneous contrasts. One noise term, ξ (dichotomous), causes the discrete transitions between a bump state (with the value of 1) and an inter-bump state (with the value of 0.02). A second noise term, η, is an exponentially correlated Ornstein-Uhlenbeck (OU) process with auto-correlation time of 1 s. This OU produces the contrast variability seen within contrast bump intervals. The instantaneous contrasts calculated from the recording (dashed lines) and representations of the model (solid lines) are shown in three cases: **(A)** mean(BD) = mean(IBD) = 4.4 s; **(B)** mean(BD) > mean(IBD); and **(C)** mean(BD) < mean(IBD). The parameters A and σ are the mean contrast value and the strength of the OU process η; here they take values of 0.2 and 0.1, respectively.

Mean BD and mean IBD vary broadly in interactions between Rfish and Ffish (Figures 6B,C). We can now design artificial time series for instantaneous contrast statistics similar to those seen in interactions between most fish pairs. Three sample realizations of time-varying contrast are demonstrated in Figure 8 with different mean BDs and mean IBDs, along with the contrasts arising from a real interaction.

## Discussion

In this study, we show that the dynamic contrast (or the second envelope, E2) of natural signals received by weakly electric fish reflects the motion of neighboring fish by analyzing simultaneous electrical and video recordings of multiple pairs of fish. Specifically, the instantaneous contrast is anti-correlated with the time-varying inter-fish distance when two fish are in close proximity. Further, the contrast seen on longer time scales occurs as a sequence of stochastic bumps triggered by the random looming and receding visits of the freely swimming fish. The diverse interactions of conspecifics produce distinct characteristics of natural contrast (e.g., mean, standard deviation, contrast BDs). We then propose a stochastic model to simulate movement-generated contrasts with similar characteristics to those measured in our experiments.

Our study demonstrated that, in the context of two weakly electric fish, the major factor leading to the varying contrast of EOD-based signals received by a fish (the observer) is the distance separating the fish, as shown in Figures 1C, 2. This distance determines the average values of the dynamic contrast (Figure 3A). Different patterns of swimming and associated contrast are seen to depend on the particular fish pair chosen (Figures 3, 5). We have not investigated whether these different patterns are tied more to the identity of the swimming fish as opposed to the pair *per se*—this question could be explored in future work.

Besides the inter-fish distance, there are many other factors contributing to the contrast. These include the various swimming movements (e.g., turning or tail-bending), the angles and the phases of two superimposed electric fields (Kelly et al., 2008), and the occurrence of social signals such as chirps or the jamming avoidance response (Allen and Marsat, 2018; Henninger et al., 2018; Shifman and Lewis, 2018; Thomas et al., 2018). As a result, these factors could lead to the variance of contrast from a statistical point of view (Figure 3A). The variations of the contrast are magnified with shorter inter-fish distance, as was seen in our previous study (Yu et al., 2012). For instance, weakly electric fish tend to chirp more when they are in close proximity, normally resulting in transient (~50 ms) decreases in the first envelope E1 and the contrast (e.g., Hupé and Lewis, 2008).

We note that contrast decays exponentially with distance under our experimental conditions (Figure 1C). This is different from the power-law relationships that have been fitted to experimental data for the EOD modulations due to small objects (with fractional power-law exponents e.g., Chen et al., 2005; Babineau et al., 2006). From a mathematical point of view, within a small distance scale, an exponential function (contrast vs. distance) can be approximated by a power function (contrast vs. distance), and *vice versa*. Nevertheless, the exponential relationship could be used for modeling and to generate experimental stimuli.

Our work supports the notion that contrast can provide useful information to electrolocate conspecifics and guide the behaviors of the observer fish (e.g., navigation, collision avoidance). Meanwhile, the dynamic variations, in contrast, degrade this information and suggest that fast body movements (e.g., rapid turning, bending) or chirps could be used as disruptive or cloaking strategies, perhaps to confuse a conspecific or even an electroreceptive predator. Indeed, a fast body twist produced a pronounced and rapid decrease (or notch) on the second contrast peak in Figure 2 box II. Such a change, in contrast, could mimic an approach, for example, without a change in inter-fish distance. More studies are required to determine whether fish actively use such strategies.

It is important to note that EOD-based signals recorded in our experiment will be encoded by electroreceptors located in an anterior region of Rfish. Previous studies reported that these electroreceptors can extract envelopes that contain movement information, and project them to target pyramidal cells in the electrosensory lateral line lobe (ELL) that respond to the movement envelopes (e.g., Middleton et al., 2006; Longtin et al., 2008; Huang and Chacron, 2016). Our results suggest that the sensory circuitry of a receiving fish has access to contrast envelopes, from which estimates of distance to the other fish are presumably derived. This is so in spite of the variability in contrast as a function of Td (and likely the other body markers too) observed across different swimming trajectories (Figure 3). This raises interesting questions about how the information is gathered across receptive fields to extract more precise information about the position and orientation of conspecifics.

To our knowledge, our work provides the first characterization of long-term instantaneous contrast time series as sequences of bumps with stochastic amplitudes resulting from approach behaviors (Figure 6). The occurrences of contrast bumps, representing approaches (“arrivals” of Ffish), are well accounted for by a Poisson distribution. The average inter-arrival time is 8.07 s in our experiments. The inter-arrival time of a Poisson process is memoryless, meaning one cannot predict better than the mean what the next arrival time will be based on the most recent arrival time. Such a memory-less process is a good first-order guess of the behavior at least in this experimental context; different behavioral and social contexts could result in different behavioral dynamics. Nevertheless, it will be interesting to determine the neural mechanisms that control such apparently random behavior.

Our model for long-term contrast (Equation 1) provides a reliable and efficient way to construct a naturalistic behaviorally-relevant stimulus to mimic free-swimming conspecifics in the laboratory. It could be used to study the neural responses to a moving conspecific and different approach behaviors. It can also be used to explore the multiple time scales of adaptation in the electroreceptors (Clarke et al., 2013) and the ELL evoked by a short-term stimulus (within one contrast bump) and long-term stimuli (a sequence of contrast bumps), similar to studies in the visual system (Ulanovsky et al., 2004).

While the form of the stochastic process we have chosen does justice to the main features of the signal, namely within bump variability as well as abrupt increases and decreases in overall amplitude, it is nevertheless an approximation. The electric field does decrease sharply with distance due to its dipolar nature, and this is approximated by a discrete switching process. Thus, we do not expect that the data will be well fitted by this model near the switches since there the overall signal amplitude varies continuously. The additive lowpass-filtered noise (OU process) on top of the dichotomous noise blurs out the associated discrepancies. Further, it is difficult to conceive of a simple stochastic process that would exhibit the strong nonlinearity imposed by the approximately dipolar relation between field strength and distance from the body. One could invoke a single non-Gaussian process, obtained e.g., by filtering a Gaussian process with a suitably designed or fitted static nonlinearity. One could also attempt to modify the spectral properties of a Gaussian noise process to fit the complete autocorrelation function of the signal; other kinds of noises such as fractal Brownian motions could have been used. But our goal was to provide a first picture of the interactions we observed in the context of our experiments and design a computationally simple model in terms of standard and easily implementable stochastic processes.

The close proximity of fish triggers not only dynamic contrasts but also communication signals (i.e., chirps). Preliminary results indicate that contrast bumps are temporally positively correlated with the bursting patterns of chirps (not shown; see also Allen and Marsat, 2018; Henninger et al., 2018). As chirps can also generate dramatic transient changes, in contrast, it would be very interesting to investigate the interplay among these three concurrent events, namely, the motion of the fish, the dynamic contrast and the chirps. The relative orientation of the interacting animals will also be an important factor, as well as the recent history of chirp emission.

One cannot expect purely random behavior from any animal, yet we have shown that at some level, certain features of the behavior are well-accounted for by assuming random processes—and specifically, the product of two standard noise processes. It is clear that the above results need to be submitted to other tests such as very long recordings, different tank sizes and depths, and eventually, to the case of two freely moving fish. The results of those future studies will, we hope, be contrasted to those reported here to reveal where and how pure randomness fails to explain significant features of the behavior.

## Data Availability

The datasets generated for this study are available on request to the first author.

## Ethics Statement

The animal study was reviewed and approved by University of Ottawa Animal Care Committee.

## Author Contributions

All authors contributed to the design of the experiments. NY and GH carried out the experiments and data analyses. The modeling analyses were performed by NY. NY wrote the article with input from JL and AL, who also supervised the whole project.

## Conflict of Interest Statement

The authors declare that the research was conducted in the absence of any commercial or financial relationships that could be construed as a potential conflict of interest. The reviewer JE declared a past co-authorship with one of the authors JL to the handling Editor.
